# Tier 2 adult weight management services in the UK: A case study evaluation of local authority provision of targeted services for higher‐risk groups in England

**DOI:** 10.1111/cob.12723

**Published:** 2024-12-04

**Authors:** Lorraine McSweeney, Charlotte Rothwell, Ashley Adamson, Simon Barrett, Claire Mathews, Scott Lloyd, Mackenzie Fong

**Affiliations:** ^1^ Population Health Sciences Institute Newcastle University Newcastle upon Tyne UK; ^2^ NIHR Applied Research Collaboration, North East and North Cumbria Gosforth UK; ^3^ Department of Health and Social Care Office for Health Improvement and Disparities London UK; ^4^ Public Health South Tees, Middlesbrough Council and Redcar & Cleveland Borough Council Middlesbrough UK; ^5^ School of Health and Life Sciences Teesside University Middlesbrough UK

**Keywords:** tier 2 services, high risk groups, weight management services

## Abstract

In 2021, the UK Government announced additional funding in England for Adult Weight Management Services (AWMS); it was specified that the extra funding must be used to commission or extend existing tier 2 services. The Office for Health Improvement and Disparities encouraged commissioners to prioritise services for higher‐risk groups such as those with learning disabilities, severe mental illness, people from minority ethnic groups, those living in deprived areas and men. To better understand the findings from previous survey work and to explore the implementation of targeted services in greater depth, we undertook a multiple case study comprising eight tier 2 adult weight management service providers and 35 individual stakeholder interviewees. Using the Consolidated Framework for Implementation Research as an interview guide and in data analysis, we determined key enablers and barriers to successful service provision and programme implementation. Good practice for successful AWMS provision for higher‐risk groups includes, having an existing programme in place that can be adapted, ensuring adequate time for programme development/implementation, having good existing networks/partnerships, collaborative working and putting the target group at the heart of any intervention. The findings from this work provide practical recommendations for policy and practice when targeting tier 2 services for higher‐risk groups.

**What is already known about this subject**

- UK guidelines recommend provision of local behavioural weight management (tier 2) services for adults living with overweight and obesity.
- Local authority commissioners were encouraged to use additional government grant funding to prioritise tier 2 services for higher‐risk groups in their local areas.
- A proportionate universal model that targets more disadvantaged groups could help reduce disparities in obesity across the socio‐economic gradient.

**What this study adds**

- This case study evaluation provides insight into the challenges faced by local authorities and relevant stakeholders in implementing targeted adult weight management services for higher‐risk groups.
- Good practice for successful targeted adult weight management service (AWMS) provision includes already having an existing programme that can be adapted, ensuring adequate time for programme development/implementation and having good existing networks/partnerships and collaborative working.
- Services should be developed and ideally, co‐produced with relevant stakeholder/community groups and service users, thus, putting the targeted group at the heart of any intervention.

## 1 INTRODUCTION

In 2020, the UK government announced their new obesity plan, which included enhancing weight loss support for people living with obesity.^1^ This included an NHS digital weight management programme, which is an online 12‐week behavioural and lifestyle programme, offering free online support via GP and primary care referrals working alongside existing weight management services.^2^ Also in 2021, the Adult Weight Management Services (AWMS) grant worth £30.5 million was introduced in England,^3^ herein referred to as the ‘AWMS Grant’. The additional funding, which was only available for one year,^4^ was distributed by Public Health England (PHE) as an executive agency of the Department of Health and Social Care in England with the remit to protect and improve health and well‐being and reduce health inequalities. The AWMS Grant was in addition to public health funding already available to local authorities (LA), and it was specified that the extra funding must be used to commission new or expand existing tier 2 AWMS.^4^ Tier 2 services are behavioural weight management programmes that are considered the first‐line treatment for those living with excess weight. They are generally delivered in community settings and aim to promote weight loss through providing support for healthy eating, physical activity and behaviour change. This initiative was proposed to be part of a place‐based, whole systems approach to tackling obesity and promoting healthier weight.^4^ Whilst provision for specific population groups was not required, PHE (disbanded and responsibilities for diet and healthy weight transferred to the Office for Health Improvement and Disparities (OHID) from October 2021) encouraged commissioners to prioritise services for higher‐risk groups^5^ that experience greater rates of living with obesity and additional barriers to engaging with AWMS. These groups included: men, people living in more deprived areas, people from minority ethnic groups, people living with severe mental illness (SMI) and people living with physical and/or learning disabilities. A proportionate universal model whereby AWMS are targeted to more disadvantaged groups could help reduce disparities in obesity across the socioeconomic gradient.^6^ LAs had between April 2021 and March 2022 to accept and spend the AWMS grant allocation. LAs were required to provide OHID with data on AWMS provision and ensure that the AWMS providers reported a minimum data set of anonymised service user level data^3^ (quarters 1&2 published Jan 2022).^7^ Whilst the minimum dataset provides information on characteristics of those who were referred, attended, and completed an AWMS, it does not provide details of the individual services. LAs were notified that this new AWMS grant was not going to be renewed for the financial year 2022/23 because funding was reprioritised to fund the UK government's Living with COVID‐19 Strategy.^8^

We previously published a cross‐sectional survey study involving an environmental scan of LA‐commissioned AWMS^9^ to understand what provision was available, who could access the services and to understand the barriers and enablers to commissioning and providing the services. While this survey provided a broad insight from tier 2 AWMS providers, we were unable to explore the implementation process in‐depth. Several survey respondents (AWMS providers) also reported the need to better understand provision for higher‐risk groups. Numerous studies have explored the efficacy of tier 2 weight management interventions set in trial settings^10^; however, studies reporting how services are being delivered on the ground are scarce. A recent study^11^ conducted qualitative interviews with commissioners to explore implementation of tier 2 AWMS in the York and Humber region. While this study provided useful insights and recommendations for the commissioning process, the perspectives of service providers and other stakeholders were absent. Capturing the perspectives of the those who actually deliver the services and their experiences of doing so is an important element to help understand more fully what is happening on the ground.

Therefore, the aim of the current study was to build on our survey findings, and previous research by exploring with commissioners and providers of AWMS in‐depth aspects of service provision, development and processes that may help or hinder implementation of tier 2 AWMS for higher‐risk groups.

## 2 MATERIALS AND METHODS

### 2.1 Design

A multiple case study design; case studies are a good way to explore how services are delivered in a real situation.^12^ The use of a case study methodology can be valuable to understand why interventions work, or do not work, in real life. Interventions usually depend upon the involvement and engagement of several stakeholders in a bound situation, for example, an organisation.^13^

### 2.2 Case studies and participants

Case studies were LA commissioned tier 2 adult AWMS providers in England that catered to higher‐risk groups as identified by OHID, i.e., men, people belonging to ethic minority groups, people living with a severe mental illness (SMI), people with a disability(s) and people living in areas of greater deprivation. We recruited eight AWMS providers in England as case studies. Within each case study, we recruited groups of key informants/stakeholders including commissioners of the service, and service staff including service provider managerial staff and frontline deliverers and trainers.

### 2.3 Theoretical lens

As we were concerned with the barriers and facilitators of service implementation, this work was informed by the Consolidated Framework for Implementation Research (CFIR).^14^ The CFIR provides a practical structure and an organisational framework for exploring implementation of innovations (i.e., AWMS) in complex and transient contexts. It helps to generate knowledge around what works, where and why in different settings. It is composed of five major domains: outer setting, inner setting, characteristics of the individuals involved, intervention characteristics and the process of implementation.^14^ The CFIR was used in data collection (i.e., interview questions) and analysis.

### 2.4 Recruitment and inclusion criteria

Sampling and recruitment of potential case studies was informed by responses to our previous survey^9^ and local knowledge and insight from appropriate local stakeholders, e.g., public health practitioners and commissioners of tier 2 AWMS. Our focus was on those service providers who were offering targeted services. We also considered the urban/rural geographic location of case studies and aimed to recruit from several England regions, and the likely ability to achieve the depth of insight required.^15^

Potential services that were identified by local contacts, who gave consent and showed willingness to be approached were contacted by researchers. The researcher contacted the service provider via email to ask if their service would like to take part in the evaluation. A participant information sheet was provided so that participants understood what was involved in the evaluation. Key informants/stakeholders within each case study were identified (commissioners, service providers, provider managers and programme deliverers/trainers) using a purposive and snowball sampling strategy^16^ and approached via email to ask if they would be willing to be interviewed.

Those who agreed to an interview were asked to complete an online consent form. Consent was also taken verbally prior to each interview. Interviews took place either online (Teams) or by telephone, dependent on the preference of the participant. The interviews were audio recorded using the Teams online function or a voice recorder. Files were sent to a university approved transcription company to be transcribed verbatim and recordings were deleted.

### 2.5 Interview guide development

The interview topic guide development was guided by the findings from our previous survey,^9^ the CFIR^14^ (Supplementary material) and reviewed by our public involvement members. Questions were tailored to be relevant for the different stakeholder group, i.e., commissioners, service providers, provider managers and programme deliverers/trainers. Interviewees were asked questions relating to elements such as intervention characteristics, intervention goals, meeting service user needs, external/internal support and communication.

### 2.6 Data analysis

Transcripts were coded using constructs from the CFIR.^14^ Each case study was analysed as an individual case, and data from each case were cross‐referenced in a matrix table and, using framework analysis,^17^ summarised to provide overall analytic themes. Framework analysis is increasingly used in health research and allows the ability to compare data across cases as well as within cases.^17^ Further analysis of the CFIR code summaries and framework themes identified barriers and enablers of service delivery from the perception of each stakeholder group.

### 2.7 Ethics

Ethical approval was granted by Newcastle University Faculty of Medical Sciences Research Ethics Committee (Ref: 2401). Before participating in the study, all survey respondents provided their informed consent.

## 3 RESULTS

Eight tier 2 AWMS were recruited. Across these eight cases, 35 individual stakeholders took part in an interview. Interviews lasted approximately 30–60 min. Table 1 outlines the case study areas, the type of service provider and the number and roles of participants interviewed within each case study. The majority of case study services were located in the North East of England, four providers were LAs, with two providing in‐house services, three were social enterprises and one a commercial provider. The majority of AWMS were targeted and included AWMS which catered specifically at people with learning difficulties, men, SMI or ethnic minorities. Two of the provider programmes were not commissioned directly through the AWMS grant but did have a focus on targeted services, so were included. Two of the providers had links with voluntary, community and social enterprise (VCSE) programme champions (linked to adults with learning disabilities (x2) and an Islamic group), some of whom took part in an interview.

**TABLE 1:** Case study area, type of service provider and number and roles of participants.

ID	Case study area	Type of service provider	Targeted groups	Provider staff (managerial) (*n*)	Commissioners (*n*)	Trainers/programme deliverers (*n*)	Programme champions (*n*)
01	North East	LA in‐house	Learning disabilities	2	0	1	5
			Islamic group				
02	North East	Social enterprise	Learning disabilities	2	1	2	0
			Carers				
			Ethnic minorities				
			Severe mental health				
03	National	Commercial	Learning disabilities	2	3	1	0
			Member‐led community groups				
04	North East	LA in‐house	Learning disabilities	2	0	0	1
05	North West	Social enterprise	Ethnic minorities	2	0	2	0
			Disabilities				
			Men				
06	North East	LA	Ethnic minorities	1	1	1	0
			Disabilities				
07	South East	Social enterprise	Severe mental health	2	2	0	0
08	South East	LA	Learning disabilities	1	1	0	0
Total				**14**	**8**	**7**	**6**

### 3.1 CFIR domains

The main findings are organised by the five CFIR domains, which were used as the basis for our interview topic guide and coding framework (Table 2). We have included the CFIR construct in parentheses after each finding with illustrative quotes and have provided points for consideration when implementing AWMS. Being adaptive, having good links and relationships with other organisations/partners, understanding the needs of service users and time for planning were highlighted as key ingredients for implementing an effective AWMS.

**TABLE 2:** Example findings organised by CFIR domain with illustrative quote and points to consider for future implementation of AWMS.

CFIR domain	Finding (CFIR construct)	Illustrative quote	Points to consider for implementation
Intervention characteristics	Having an effective programme already in place was conducive for adaptation of materials/resources for targeted groups. (Adaptability)	*“The team also developed a bespoke programme for people with learning disabilities in partnership with [name] charity. So it was fully bespoke and codesigned by them, so service users, [charity] staff. So that programme got developed from there” (ID01: commissioner)*.	If an existing programme is being adapted for a higher‐ risk groups, allow adequate time, planning and ideally use co‐production methods with the community/group in question.
Outer setting	The provider had links with several partners, volunteers, GP practices and a range of community partners including schools. (Cosmopolitanism)	*“A main aim of the programme format was that the programme be delivered by partners in the community by the community, therefore, the providers established/had links and worked with a range of professionals and voluntary sectors” (ID02: provider manager)*.	Before planning a programme/intervention, explore contacts/links/networks/local communities. Who can provide expertise, advise, be a champion or collaborate?
Inner setting	Stakeholders felt very strongly about making their programme accessible to everyone and identified where there were gaps in the service. (Tension for change)	*“If we're supposed to be making information and resources available to everybody, then there always has to be an option for people with a learning disability and autism. And if there isn't an option, it's arguably discriminatory” (ID01: programme champion)*.	Consider which groups in the community are not able to access a AWMS programme. How will you convince others/management that there is a strong need for this type of intervention?
Characteristics of individuals	Trainers/programme deliverers understood the need to make service users feel welcomed and relaxed. Many stakeholders spoke of the anxiety some service users face in attending a AWMS programme and for the need to be supportive and empathetic. (Other personal attributes)	*“You've got to be empathetic, you've got to be confident, you're stood in front of 30 people, you've got to be energetic, enthusiastic around what you do, provide them with lots of different examples, different ways to deliver it, because it's really difficult to change your lifestyle, at the end of the day. I think fun as well is a good one because if you make it fun and engaging, they're [service users] going to want to come every week” (ID05: programme deliverer)*.	A programme deliverer who can create a safe, welcoming environment and understand service users' needs is likely to keep service users engaged. Having programme deliverers who live in the same community as the service users is reported to be effective.
Implementation process	Building new relationships with external groups/communities takes time. Forming a new priority group from scratch is challenging. (Planning)	*“Having that little bit more of a development and planning time at the very beginning would have allowed us to get out into the health networks and almost start the ball rolling with GP referrals rather than that become something that we did probably halfway through into the delivery period of the programme” (ID05: provider manager)*.	Commissioners highlighted difficulties with the timescales imposed with the grant funding. At least 3 months is required for programme development (especially with targeted groups who may require longer lead in time to build up trust, resources etc.

Six common themes were identified across the case studies (Box 1).

BOX 1 **Themes identified from all case study data**

- Having an effective AWMS intervention/programme in place at the time the grant was awarded helped when adapting AWMS services for targeted groups
- Allowing time for outreach work and programme development (understanding the specific needs of the services users from targeted groups)
- Short timeframe for funding and programme operationalisation
- Ensuring meeting venues are accessible/in community settings
- Programmes incorporate other aspects of behaviour change and do not just focus on weight‐loss
- Peer support for service users is an important element for ongoing sustainability

### 3.2 Barriers and enablers

To understand the elements that may contribute to good practice and effective service provision, the data were further examined by the researchers to identify barriers and enablers within each stakeholder group. Table 3 highlights the most frequently identified barriers and enablers across the case studies. No commissioners were available for interview in case study 05. Trainers/programme delivers were less likely to report barriers; however, some barriers reported by one person are highlighted. In two of the case studies (case study 01/case study 04), staff members/champions of community groups involved in the implementation of the AWMS programmes were interviewed. No specific barriers were highlighted but enablers were identified. Example quotes are provided to illustrate identified areas and labelled with case study (CS) IDs.

**TABLE 3:** Common barriers and enablers across all case studies by individual stakeholder perspectives.

Stakeholder	Barriers	Enablers
Service provider perspectives	Planning/implementation time consuming/not enough time Gaps in current model for targeted groups LA commissioning process can be complex Self‐referral process time‐consuming and not appropriate for all high‐risk groups such as those with learning disabilities	Close working/partnerships with partners Established relationship with community group/organisation Tap into existing community groups/organisations Constant monitoring and evaluation of programme Allow sufficient time for collaboration and planning Existing network of trainers Use of evidence‐based guidelines and techniques Time and effort given for promotion/recruitment GP recommendation/close links with GP/primary care Brand identity aids with service user recruitment
Commissioner perspectives	Available resources limit programme spaces In‐house/LA funding and capacity limited Timing of grant and delivery very tight COVID‐19 was a competing priority/barrier	In‐house team has control of resources Programme values and aims compatible with target/community group Use of evidence‐based guidelines and techniques Use of multidisciplinary team Good links/partnerships with community groups/organisations Learn from the process and pass learning onto the system The chosen provider was fit for purpose and produced quality over quantity Grant money enabled new groups to be targeted
Programme deliverer/trainer perspectives	Venues/venue resources/facilities not always fit for purpose Collection of MDS data onerous/time consuming High drop‐off rates Programme manuals not being ready or containing errors	Programme materials/delivery easily adapted for different groups Confidence to deliver sessions to a range of groups/people Trainer needs to be personable and approachable Trainer needs to be passionate about their role Trainers well supported by management, materials/resources and training opportunities Trainer part of a franchise and receive guidance on programme delivery/content Trainer has good links with local health care professionals Trainer will make personal contact with service users following registration and maintains communication over course
Community champions perspectives	N/A	Good partnership/working with provider/programme deliverers/local organisations Taking ownership of the project/demonstrating passion Advocating for target group

### 3.3 Service provider perspectives

For providers, the short timeframe for working with commissioners and other stakeholders and the time needed to develop a targeted service was a significant barrier for providing the type of service required:

*‘I think we probably would have been hoping that they [commissioners] would be able to spend a fair bit of their time on some of that more developmental work with the community groups side of what we were hoping for them to do, just literally the processing of just the referrals and keeping up with trying to fill those courses and everything ended up, I think, taking up the majority of the time. So there was less time available for that more community development work that we wanted them to do’ (CS06)*.

Having an existing network of collaborators and partnerships or taking time to engage with communities to nurture or build relationships was key for the development of a targeted service:

*‘We tried to build a picture of where we want the service to target and the types of population groups that we'd want them to engage and work with in the communities and the community groups that served them, to work with them to develop a bit of a bespoke offer for those particular groups in communities’ (CS03)*.

Many providers also built upon or worked towards establishing good relations with local GP practices and health care professionals to aid programme promotion and referrals:

*‘We basically communicated with all health professionals, key stakeholders across [region] that if you've got a patient that you'd like to speak about weight management to – and tier 2 is more likely going to be a feasible option – here is all the information that you need to have that conversation, and how you would direct that patient to be able to access that service. Self‐referral is quite a pure term, but obviously, GPs and health professionals still need to direct…’ (CS03)*.

### 3.4 Commissioner perspectives

Some of the case study commissioners worked in LAs often in small public health teams with many competing priorities. Those who worked with an in‐house model often found resources and capacity to be limited, which could impact upon the monitoring and follow‐up of programmes:

*‘I think if I had more time, or if all of us in public health had more time, we probably would have done a bit more hands on work with the provider to really monitor over time the take up, the demand, the challenges, the barriers, holding them to account a little bit more in relation to some of the outcomes and aspects of the specification’ (CS02)*.

However, it was also reported that working to an in‐house model could also provide staff with more control and autonomy of a programme:

*‘Yes. I think we are very lucky that we've kind of had free rein to get the resources. I think your most valuable resource is your individuals delivering the sessions and having that expertise there [in‐house]. So yes, we've not only had the resources, but we've had expertise to build, to have your on‐the‐day resources delivering the programmes’ (CS01)*.

The additional grant money enabled commissioners to reach out to different communities and groups that may not previously been able to access AWMS:

*‘So it [service provision] sort of ramped up and we expanded and tried a few different things… So we did trials and workplace pilots, and community pilots, so we were asking them [providers] to specifically try and work with more community groups and see if they could either work with those community groups to deliver the service with them or adjust the programme to meet their needs’ (CS06)*.

The use of existing guidance such as the National Institute for Health and Care Excellence (NICE) guidelines provided a basis for existing universal AWMS, but it was acknowledged that adaptations for specific groups were required:

*‘I think because we've used the NICE, we've also been really aware of now because, obviously, through Public Health, when they wanted us to do the targeted work, we've had to adapt and tweak that to suit the needs of the participants attending’ (CS01)*.

### 3.5 Programme deliverer/trainer perspectives

One of the reported barriers for programme deliverers was the issue of finding and working in venues/community spaces (especially since the COVID‐19 pandemic) that need to be fit for purpose. The cost of venues had also reportedly increased since the pandemic, which caused a barrier when considering financial resources:

*‘Whenever we're sourcing a venue, or our current venues, we aim for every session 60 to 70 members, with about 40 staying to the session. So, it would need to be a big enough venue to have 40 chairs out…., a table for the weigh desk, the new member area. So, sort of, like a village hall size, or a school hall sized venue, rather than a little committee room. Do you know what I mean? It has to be big and spacious’ (CS03)*.

The practicalities of collecting data as part of the mandatory data collection required by the commissioners and OHID from service users could be a time‐consuming activity:

*‘So it was quite an onerous, probably, for want of a better word, data‐collection form to make sure we had all of the data we needed and needed for the commissioners’ (CS02)*.

It was evident that those interviewed were enthusiastic about their role engaging with and helping service users to achieve their goals. Participants spoke of the qualities required to be a programme deliverer and the need for empathy and compassion especially towards service users who may have attendance anxiety. Services often contacted the service users prior to them attending the programme to help alleviate some of this anxiety to make sure that they knew what the programme was and to ensure they were able to commit to the programme:

*‘A big thing that we've found across the three years is that kind of… anticipatory anxiety. It's huge with this sort of course that somebody will sign up to it with all the best intentions of coming to it, but they might turn up, park in the car park and be overwhelmed at what they're about to do, not knowing anyone, not knowing what to expect, and they might just turn their car away and go home… if you can make them feel welcome, that's much better than being able to tell them about how many carbohydrates is in a bag of pasta’ (CS05)*.

Supporting service users on their weight‐loss journey includes taking account of the service users' lifestyle, home contexts and in some cases, whether they use carer support. Ensuring full support for the intended lifestyle changes can sometimes be challenging. In some circumstances, carers also require a change in behaviour, e.g., shopping and cooking habits.

*‘We also ask carers to come along, and sometimes we have to challenge that, too. If a carer comes along, we want them to come along and sit in the group environment. You do sometimes, say, see that they want to drop them off and sit in the car. Or, if there's a coffee shop or something next door, they'll drop them off. In a way, it might be a break for them, but then they're not getting enough out of the programme as we want them to, because we need them to have their carers with them…. Who will then help with shopping, and cooking and that kind of thing. That in itself is challenging because it's just making carers understand. We're very clear, as part of our triage, that “We need you to come along, as well, and be a part of things”’ (CS03)*.

Maintaining good communication links with the service users/groups was key to enhancing service user engagement. The peer support element between group members was highlighted as a positive for sustaining the weekly commitment:

*‘He [programme leader] will speak to everybody on the phone and then he'll start up a WhatsApp group a week before week zero. His communication is very much pivotal to our recruitment and those that then see the programme through’ (CS05)*.

*‘It also, to me [programme deliverer], looks like being able to continue and benefit from that peer support. So, either not necessarily weight related, but being able to engage with the community that they're [service users] living in, so joining community groups, being able to share their benefits with others, and their knowledge, and their experiences’ (CS05)*.

### 3.6 Community champions

The VCSE community champions emphasised the importance of good partnership working and collaboration with commissioners and providers and building rapport to create positive relationships to enhance the AWMS:

*‘Sometimes you have sessions where you get different instructors, different sessions you get different people, so you don't really build that rapport, whilst with the [name of programme] we had the same instructor over and over again, and [service users] made that connection with them. So rather than them being a teacher/student, it's more like a friendship… that rapport's so important, because they understood the people they were working with, and the people understood their instructor. That really worked very well’ (CS01)*.

The close working relationships the community champions had on behalf of their community allowed them to take ownership of the programme and help it progress in the best way for their community:

*‘I think what we found was, especially going back to the [name] community, they would use their own methods, finding out, for us [programme deliverer], getting them [service users] to use their WhatsApp. That's their method of communication. They would then take ownership and say, “Right, has everybody remembered there's a session today, you're coming at such and such a time?”… I mean, the [name] community had a collaboration event once the programme was finished and they invited everybody that had been involved in the programme… and we had 150 people attend. The Director of Public Health, she came to that’ (CS01: programme deliverer)*.

## 4 DISCUSSION

Findings from this case study research aimed to provide a greater understanding of findings from our survey^9^ with tier 2 AWMS providers and explore in‐depth aspects of tier 2 AWMS service provision, development and processes that may help or hinder implementation. We examined the perspectives of service commissioners and staff who support service delivery including trainers/programme deliverers and community groups/champions.

Whilst the case studies varied with regard to location, type of organisation and model of delivery, length of time in operation and commissioning processes, there were several key themes across all AWMS with respect to barriers and enablers to effective service provision. All the case studies used well‐known established evidence to underpin/develop their programmes such as the NICE Guidelines^18^ or behaviour change theory models. The additional AWMS grant funding allowed providers to either set up a new service, extend an existing service, or adapt an existing service to target higher‐risk groups. Our focus for this study was providers who were offering tier 2 adult AWMS for targeted higher‐risk groups as identified by OHID, i.e., men, adults with learning disabilities, minority ethnic groups, those from low socio‐economic groups, and those with severe mental illness. Service users in certain higher‐risk groups are less likely to remain engaged with a AWMS programme^19^ and attrition can be high. It is also reported that those with poorer mental health and/or severe anxiety are at greater risk of attrition.^19^ Stakeholders in this interview study highlighted that not only are those with anxiety less likely to continue with a programme but may also require several attempts to initially attend a session and may even struggle to get out of the car in the venue carpark. Providers recognise these potential barriers and have attempted to address attendance anxieties with strategies such as providing one‐to‐one contact prior to the first session and/or sending videos showing the venue, entrance, and session room.

Working in partnership with communities, other organisations and groups were repeatedly highlighted as being key for establishing links and determining the needs of a community or targeted group. The providers in one of the case studies in the present study had established links with a local VCSE charity that supports adults with learning disabilities. Working with this VCSE group, the providers were able to co‐produce and adapt an existing programme specifically for the needs of the service users, which has been shown to be effective.^11^ This partnership also provided additional support for the service users when in ‘external environments’, which Spanos et al. report as a potential barrier for healthy eating practices.^20^ Whilst not all the providers in the case studies were able to co‐produce a programme with people from the target group due to time or capacity, it was acknowledged that allowing time for collaboration and planning, identifying who can provide expertise, advice, be a champion or join forces in service/programme development is likely to lead to a more effective delivery. A co‐production model including all stakeholders (those with lived experience, their carers, communities and health professionals) offers potential for knowledge transfer into practical application.^21^

Moreover, having good relationships with local GPs and healthcare professionals to assist with programme promotion and referrals was considered a key aspect for successful service delivery. Those providers that prioritised strategies to engage with GP practices spoke of the benefits of doing so. Having excellent communication between providers and referrers has been described as fundamental^22^ and needs to be a two‐way process with both referrers (such as health professionals) and providers engaging as it can pose challenges for providers if dependent on these types of referrals alone.^22^ Moreover, it has been reported that GPs can be reluctant to raise a weight management conversation with a patient due to limited consultation time or uncertainty on how best to approach the conversation.^23^, ^24^, ^25^ Most providers in this study also encouraged/promoted self‐referrals, whilst this pathway was believed to be effective, for some, it could be time‐consuming to process and not suited to all service users in certain higher‐risk groups, e.g., those with learning difficulties.

It was evident that having the ‘right’ programme deliverer/trainer of sessions would hugely impact the likelihood of service users remaining in a programme. Providers ensured trainers received adequate training and monitoring, giving support and feedback where necessary. Trainers were encouraged to take ownership of the delivery of the sessions and provide a service user‐centred approach. Building trust and rapport with service users and developing their confidence are a few of the essential qualities trainers are expected to exhibit.^22^ The trainers we spoke to were passionate about their roles and enjoyed seeing service users grow in confidence and self‐belief. However, some trainers sometimes faced barriers in service delivery. They spoke of programme manuals initially not being ready, containing errors, difficulties in accessing venues or not having the right resources/equipment in place; this may in part be due to the short timeframes in which commissioners/providers had to implement the grant and programmes.

The additional funding through the AWMS grant, although limited to one year, was welcomed and provided opportunities for a more bespoke, targeted service for high‐risk groups, who would not normally find universal offers accessible. However, the short timeframes for accessing the additional funding and develop and implement the programme was a barrier reported by many stakeholders and was also reported in the survey data.^9^ Indeed, two of the case studies stated having to return the AWMS Grant as they were not able to deliver a service in the timeframe available. Time‐limited funding places pressure on public health and AWMS professionals and resources and can perpetuate an ineffective cycle of service commissioning and decommissioning.^26^ Our findings also suggest that increased funding alone is not sufficient to achieve successful service delivery,^9^ and providers need time to engage with the communities they serve.

Additionally, service users who use carer support are encouraged to have their carers attend each session with them. However, effective carer engagement was perceived to be a barrier for some stakeholders, with some carers using the sessions as ‘time‐out’ or not being supportive of suggested shopping, cooking and eating changes for their client. In a study by Spanos et al., (2012) exploring the perspectives of carers of adults with learning disabilities,^20^ it was reported that family carers may struggle to find time to support their family member as well as dealing with their own busy lives. Paid carers too reported that staffing levels and multiple carers working with a client acted as a barrier for supporting positive dietary changes.^20^

### 4.1 Comparison of interview findings with previous survey findings

As discussed, the qualitative case studies were designed to further explore and provide context to our previous survey of tier 2 AWMS providers.^9^

Survey findings included:

- Tier 2 services were similar in terms of their duration, format and mode of delivery; however, there was variation in participant eligibility criteria and programme content between and within services. Several programmes provided support for other health and well‐being issues such as debt assistance, mental health and smoking cessation.
- To improve future AWMS commissioning and delivery, AWMS providers need to be allowed adequate time and resources to properly prepare for service delivery. Referral systems and criteria should be made clear and straightforward to both referrers and service users. Strategies to manage surplus referrals should be explored to reduce the likelihood that participants are referred inappropriately.

What this case study adds:

- Further highlights the importance of adequate time and resources for service development and delivery.
- Services should be developed (and ideally) co‐produced with relevant stakeholder/community groups and service users.
- Building and maintaining good relationships/communication with other organisations such as GP practices and other partners is reported to enhance the referral process.
- Having a baseline programme that can be tailored to specific needs/groups is advantageous.
- Programme venues/locations and timing of sessions need to be flexible and accessible.
- Pre‐programme attendance support such as one‐to‐one contact and/or videos showing the venue, building entrance, session room and trainer/programme deliverer are reported to be helpful.
- Having the same programme trainer/deliverer at each programme session allows rapport and trust to be built.
- The peer support element of the face‐to‐face format is especially valued for service user engagement and programme sustainability.

### 4.2 Recommendations for policy and practice

These findings can help with future planning and implementation of commissioning and provision of adult AWMS, especially with high‐risk groups. Box 2 highlights recommendations identified for effective targeted programme implementation.

BOX 2 **Recommendations for targeted AWMS for policy and practice**

- If an existing programme is being adapted for a higher‐risk groups, allow adequate time, planning and ideally use co‐production methods with the community/group in question.
- Before planning a programme/intervention, explore contacts/links/networks/local communities. Who can provide expertise, advise, be a champion or collaborate?
- Consider which groups in the community are not able to access a AWMS programme. How will you convince others/commissioners/funders/management that there is a strong need for this type of intervention?
- A programme deliverer who can create a safe, welcoming environment, create peer support opportunities and understand service users' needs is likely to keep service users engaged. Having programme deliverers who live in the same community as the service users is reported to be effective.

### 4.3 Strengths and limitations

This study makes a novel contribution to the literature by generating evidence on the implementation of tier 2 adult AWMS in real‐life context. While these services are commonly commissioned across the country based on studies evaluating their effectiveness in trial settings, there is a stark lack of evaluations exploring their implementation ‘on the ground’. The use of case study methodology allowed us to compare and contrast experiences of and barriers and facilitators to commissioning and implementing tier 2 AWMS across a range of contexts and from a range of stakeholder perspectives. It should be acknowledged, however, that the findings are based on the stakeholders who agreed to participate. Had we spoken different stakeholders, we may have received some differing views. Despite this, the range of providers/stakeholders that did agree to participate provided us with rich data outlining potential barriers and enablers to successful tier 2 AWMS provision.

## 5 CONCLUSIONS

Good practice for successful AWMS provision for higher‐risk groups includes having an existing programme in place that can be adapted, ensuring adequate time for programme development/implementation, having good existing networks/partnerships, collaborative working and putting the target group at the heart of any intervention.

## AUTHOR CONTRIBUTIONS

MF and CR conceived the study. MF, CR and LM conceived the study design. CR and LM collected the data. CR, LM, MF and SB analysed the data. All authors were involved in writing the paper and had final approval of the submitted and published versions. The authors would like to thank colleague Sarah Richardson for assisting with data analysis checking, and all the participants who agreed to take part. We also acknowledge the ongoing support of the wider National Enhanced Service Incentive Evaluation (NESIE) project team lead by NIHR Applied Research Collaboration Oxford and Thames Valley.

## CONFLICT OF INTEREST STATEMENT

The authors declared no potential conflicts of interest with respect to the research, authorship and/or publication of this article.

## Supporting information


**Data S1.** Supporting Information.
