# Supplementary material for: Tier 2 adult weight management services in the UK: A case study evaluation of local authority provision of targeted services for higher‐risk groups in England
Source: Clin Obes. 2024 Dec 4;15(2):e12723. doi: 10.1111/cob.12723 (PMC11907090; doi:10.1111/cob.12723)
Supplement: Supplementary file 1 — Data S1. Supporting Information. [file COB-15-e12723-s001.pdf]

Tier 2 adult weight management services in the UK: A case study evaluation of Local Authority provision of targeted services for higher-risk groups in England

*^Lorraine McSweeney[1], ^Charlotte Rothwell[1,2], Ashley Adamson[1], Simon Barrett[1,2], Claire Mathews[3], Scott Lloyd[1,4,5], Mackenzie Fong[1,2]

[1] Population Health Sciences Institute, Newcastle University, Newcastle upon Tyne, UK

[2]NIHR Applied Research Collaboration, North East and North Cumbria, UK

[3]Department of Health and Social Care, Office for Health Improvement and Disparities, UK

[4]Public Health South Tees, Middlesbrough Council and Redcar & Cleveland Borough Council, Middlesbrough, UK

[5]School of Health and Life Sciences, Teesside University, Middlesbrough, UK

Evaluation of targeted Tier 2 AWMS
Semi-structured interview topic guide:

What factors inhibit and facilitate successful referral to and retention in LA-commissioned tier 2 WMS for priority groups?

Introduction

Thank you for agreeing to talk with me today. As outlined in the study information sheet, we are going to talk about Tier 2 weight management services, [*name of service*], your role within that and your thoughts on what works well, what doesn't work as well, and why. The interview should take between 45 minutes to an hour, and you are free to stop the interview at any time if you decide you no longer wish to take part. Can I check you've read the information sheet and you're happy to proceed?

Confirm e-consent has been completed
Switch on recording function

Introduction:
Can you please describe your role within [*name of programme*]?

a. Commissioner
b. Referrer
c. Programme implementer? Specifically, leader/front line deliverer/support?
d. Other?

What does this involve in your day-to-day work?

A. Questions for COMMISSIONERS

1. What provision did you have before, during and after the PHE grant?
    - **Prompt**: funding streams (specific AWMS grant and general PH grant)

How was the decision made:

2. Who is involved in the commissioning of [programme] (IS)?
    - You? Service users? Practitioners?
    - In what ways?

Why was the decision made:

3. Why do you think the decision was made to commission [*programme*] for [priority group e.g., people with learning disabilities]? (IS; TC)
    - What type of external factors influenced this decision? E.g., policies, incentives, campaigns, other areas doing similar, service goals? (EP)

Choice of intervention and intervention characteristics:

4. How did you decide which/service program to commission?
    - What kind of information/evidence are you aware of that shows that this intervention would work? NICE? Others? (ES)

Meeting target audience needs:

5. To what extent do you think [*the programme*] meet the needs and preferences of the service users? In what ways? (PN)
    - Was the intervention changed or adapted in any way to meet these needs and preferences? In what ways? (PN)
    - What barriers or challenges do service users face to participate in the intervention e.g., referral, retention? Were these addressed and if so, how? (PN)
    - In what ways was the programme communicated/promoted to service users? (IP) can you provide any examples
    - In what ways was the programme communicated/promoted to key stakeholders outside your setting/organization? (KS) can you provide any examples
    - To what extent do you think key stakeholders influenced service users to participate in the intervention? (referrals) (KS)
    - Do you think the intervention has been effective in [*setting*]? Why do you say that? (KB)
6. Are there any 'hidden/underappreciated' inequalities that impact the delivery of the service?

Meeting intervention goals:

7. Did your setting/organization set any goals related to rates of referral and retention? (GF) if yes, what was the reason behind setting these specific goals?

- What do you perceive to be a marker of success? I.e. how are you measuring success or what does success look like to you?

Implementer characteristics:

8. We are interested in the person delivering and leading the programme and trying to understand what personal attributes they may bring to the role. What do you think the personal attributes are to make a successful programme deliverer/leader to implement the intervention? (PA)?
    - Eg: motivation, background knowledge, capacity, rapport with service users

Communication, partnerships, and external support:

9. Did you have other people outside of your setting/ organization that helped with commissioning the intervention? (CA)
    - What was their contribution to the commissioning of the intervention? (CA)

10. What kind of information sharing do you have with professionals outside your setting/organization about [*programme*] e.g., GPs, PCNs, VCSE groups for target users and carers? (Co)

Competing priorities:

11. When implementing the intervention, were there any other high priority activities taking place at the same time? Describe (RP) (e.g., COVID-19 and PH team response)
- Did this affect commissioning/procurement? (RP)
- How was this managed?

Resourcing:

12. Were adequate resources provided to commission [programme]? Eg: staffing, preparation time, etc. (R)
    - Were there any incentives offered within your setting/ organization to help ensure the [programme] was implemented successfully? (OIR)
    - Were there any costs to [*your organisation*] in implementing the intervention? If yes, were any of these costs unexpected? ($)

General:

13. Can you think of any other factors that influenced the decision to commission and the actual commissioning of [service]?
14. Can you think of any other factors that influenced the implementation of [service]?
15. Can you share any examples best practice?

B. Questions for PROVIDERS (team leaders and front-line deliverers)

Warm up

1. Can you please briefly summarise [*programme*]?
- Who is it for

- How are service users referred into [*programme*]?
- What process must service users complete to enroll?
- How is it delivered?
- What are the main aims and activities?
- How is recruitment managed/monitored?
- What demographic characteristics are collected?

Intervention characteristics:

2. What kind of information/evidence are you aware of that shows that this intervention would work? NICE? Others? (ES) Are you able to give any examples?
    - How adaptable is [programme]? (Ad)
    - Is [programme] well packaged and designed? (ID)
    - Is [programme] affordable? ($)
    - Was [programme] piloted/trialed before it was rolled out? (Tr)
    - What, if any, challenges have you faced in the way that [programme] works?
3. Were service users involved in the development of [programme]? In what way?

Meeting target audience needs:

4. To what extent do you think [*the programme*] meet the needs and preferences of the service users? In what ways? (PN) Are there any gaps?
    - Was the intervention changed in any way to meet these needs and preferences? In what ways? (PN)
    - What barriers do service users face to participate in the intervention e.g., referral, retention? Were these addressed and if so, how? (PN)
    - How was the programme communicated/promoted to the target audience? (IP)
    - How was the programme communicated/promoted to key stakeholders outside your setting/organization? (KS)
    - To what extent do you think key stakeholders influenced service users to participate in the intervention? (referrals) (KS)
    - Do you think the intervention has been effective in your setting? Why do you say that? (KB)
    - What opportunities are available to service users once they have completed the programme?
5. Are there any 'hidden/underappreciated' inequalities that impact the delivery of the service?

Meeting intervention goals:

6. Did your setting/organization set any goals related to rates of referral and retention? (GF)
    - What do you perceive the be a marker of success? what does success look like to you? Examples?

Organizational/management support:

7. Is there anything about your work unit/setting/organization that has impacted on your referral and retention of participants in the intervention? In what ways? (Cu)
    - How would you describe the general willingness (receptivity) within your setting to implementing [programme]? (IC)
    - What level of support/endorsement was provided by senior management / leaders for the implementation of the intervention? (LE)
    - What, if anything, would help with future programme delivery?

Implementer characteristics:

8. What personal attributes of programme leaders/deliverers do you think were useful to recruit and retain service users into the intervention (PA)? E.g.: motivation, background knowledge, capacity

Internal support:

9. Apart from [*the implementer*] were there other people within your setting/organization who were champions of the intervention e.g., GPs, PCNs, VCSE groups for target users and carers? (Ch)
    - What was their contribution to the implementation of the intervention? (Ch)

Communication, partnerships, and external support:

10. Did you have other people outside of your setting/ organization that helped with implementing the intervention e.g., referral, retention? (CA)
    - What was their contribution to the implementation of the intervention? (CA)

11. What kind of information sharing do you have with professionals outside your setting/organization about the intervention? (Co)
    - How was the service marketed/advertised to patients and to referrers?
12. Do you think referral pathways are clear and well-integrated into your service?

Resourcing:

13. Were adequate resources provided to implement the intervention? E.g.: staffing, preparation time, space, equipment, etc. (R)
    - Were there any incentives offered within your setting/ organization to help ensure the intervention was implemented successfully? (OIR)
    - Were there any costs to your setting in implementing the intervention? If yes, were any of these costs unexpected? ($)

General:

14. Can you think of any other factors that have influenced referral and retention within [service]?
    - What if anything, would you do differently
    - What do you think worked well? Examples of best practice?

C. Questions for REFERRERS

Warm up

1. How comfortable do you feel about discussing weight loss with patients?

- If the patient's weight is not the consulting/presenting issue, how would you discuss WMS?
- How do you decide if a patient should be referred to WMS?
- How are patients' weights monitored?
- Are patients' attendance of a WMS followed up/recorded?

How decisions are made

2. Can you talk through how you decide which service/programme a patient should be referred to?
    - NHS or local services?
    - What information do you have about local services?
    - How is this information shared with you?
    - How do you decide which service/programme would be most relevant for a patient?

Intervention characteristics:

3. Can you please tell me what you know about [programme] and what involvement you've had with it?

4. What kind of information/evidence are you aware of that shows that [programme] would work? NICE? Others? (ES)
    - How does the intervention compare to other similar existing programs in your organization/setting? (RA)
    - What advantages/disadvantages does the intervention have compared to these other programmes? (RA)

Meeting target audience needs:

5. To what extent do you think [*the programme*] meet the needs and preferences of the service users? In what ways? (PN) any gaps?
    - What barriers do service users face to participate in the intervention e.g., referral, retention? Were these addressed and if so, how? (PN)
    - How was the programme communicated/promoted to key stakeholders outside your setting/organization? (KS)
    - To what extent do you think key stakeholders influenced service users to participate in the intervention? (referrals) (KS)
6. Are there any 'hidden/underappreciated' inequalities that impact the delivery of the service?

Internal support:

7. Are there people within your setting/organization who are champions of [programme], or of weight management in general? (Ch)

- How has this influenced your referral to [*programme*]?

Communication, partnerships, and external support:

8. Have you or others in your organisation worked with [*programme*] to refer to and retain within services users to and within [*programme*]? (CA)
   - What was their contribution to the implementation of the intervention? (CA)

9. What kind of information sharing and communication do you have with [*programme*]? (Co)
   - How was [programme] advertised/marketed to you?

10. Do you think referral pathways are clear and well-integrated into [programme]?

Resourcing:

11. Were adequate resources provided to facilitate referral to [programme]? Eg: training, time (R)?

General:

12. Can you think of any other factors that have influenced referral to [service]?
    - What, if anything, would you do differently?
    - What has worked well? Examples of best practice?

ALL PARTICIPANTS

1. Are you able to share any documents with us that would be useful for our project? (Examples include)

- Strategies and reports
- Performance monitoring returns
- Meeting minutes
- Work packages

2. We would like to speak to others who may be involved in the commissioning of, referral to, or implementation of [name of programme], are you able to provide me with any contacts/or introduce me to them?

3. Is there anything else you would like to share about the process/service/ programme?

4. Do you have any questions?

Thank participant for their time and switch off recording function.

CFIR constructs

Adaptability (Ad)
Available Resources (R)
Champions (Ch)
Cosmopolitanism (Co)
Cost ($)
Culture (Cu)
Evidence Strength & Quality (ES)
External Change Agents (CA)
External Policy (EP)
Goals and Feedback (GF)
Implementation Climate (IC)
Intervention Design (ID)
Intervention Participants (IP)
Intervention Source (IS)
Key Stakeholders (KS)
Knowledge & Beliefs about the Intervention (KB)
Leadership Engagement (LE)
Organizational Incentives & Rewards (OIR)
Other Personal Attributes (PA)
Patient Needs & Resources (PN)
Relative Advantage (RA)
Relative Priority (RP)
Tension for Change (TC)
Trialability (Tr)
